# The structure–activity relationship review of the main bioactive constituents of *Morus* genus plants

**DOI:** 10.1007/s11418-019-01383-8

**Published:** 2020-01-02

**Authors:** Jiejing Yan, Jingya Ruan, Peijian Huang, Fan Sun, Dandan Zheng, Yi Zhang, Tao Wang

**Affiliations:** 1 Tianjin State Key Laboratory of Modern Chinese Medicine, Tianjin University of Traditional Chinese Medicine, 312 Anshanxi Road, Nankai District, Tianjin, 300193 China; 2 Tianjin Key Laboratory of TCM Chemistry and Analysis, Institute of Traditional Chinese Medicine, Tianjin University of Traditional Chinese Medicine, 312 Anshanxi Road, Nankai District, Tianjin, 300193 China

**Keywords:** *Morus* genus plants, Bioactive constituents, Structure–activity relationships

## Abstract

**Abstract:**

*Morus* genus plants are mainly distributed in the temperate to tropical areas over the world and include 17 species and two subspecies. Due to their excellent pharmacological activity, security in food additives and high value in the national economy, *Morus* genus plants have drawn more and more attention in recent years. In the light of the references published over the last few decades, flavonoids, benzofurans, stilbenes, and Diels–Alder adducts have been reported to be the main bioactive constituents of *Morus* genus plants. This review summarizes the compounds with excellent bioactivities isolated from *Morus* genus plants as well as their structure–activity relationships (SARs), which might be useful for the further research and development of *Morus* genus plants.

**Graphic abstract:**

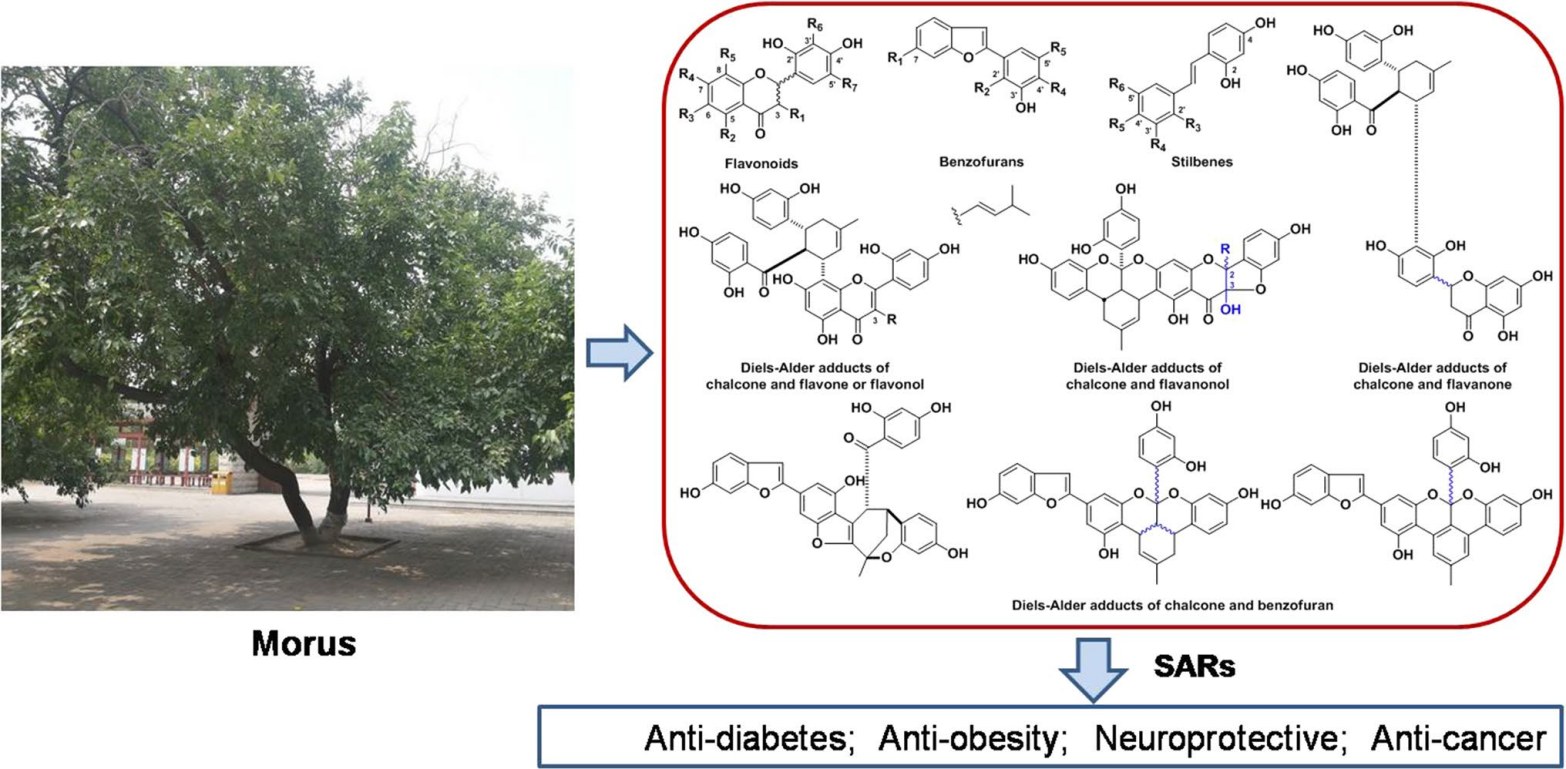

The aromatic heterocycles with excellent bioactivities isolated from *Morus* genus plants as well as their structure–activity relationships (SARs) were summarized.

## Introduction

*Morus* genus plants comprise flowering trees belonging to Moraceae family and are distributed in the temperate to tropical areas over the world. According to the webpage of https://www.theplantlist.org, the *Morus* genus comprises 17 species and two subspecies. In traditional Chinese medicine (TCM), the leaves, root, bark, stems, and fruits of *M. alba* are used for treatment of rheumatism, cough and inflammation, and the leaves and fruits of *M. alba* serve as foodstuff around the world. Recently, not only *M. alba*, but also other *Morus* genus plants’ (such as *M. alba* var. *tatarica*, *M. lhou*, *M. australis*, *M. yunnanensis*, *M. cathayana*, and *M. nigra*) chemical components and their biological activity have been evaluated [1–3].

Polyphenols, including flavonoids, benzofurans, stilbenes, and Diels–Alder adducts were considered as the main chemical constituents and the key bioactive ingredients of *Morus* genus plants. Polyphenols from *Morus* genus plants exhibit multiple bioactivities, such as anti-diabetic, cytotoxicity, hypolipidemic, anti-oxidative, anti-inflammatory, anti-microbial, anti-fungal, skin whitening [4], and neuroprotective effects [5]. Among these, the anti-diabetic effect was confirmed by different clinical research groups [6–9].

This paper intends to provide an review of biological chemical constituents present in *Morus* genus plants and summarize their structure–activity relationships (SARs) on α-glucosidase, lipase, tyrosinase, β-secretase, acetylcholinesterase and cytotoxicity, which may be benefit for nutritional supplements development and structure modification of lead compound from *Morus* genus plants.

## Polyphenols from *Morus* genus plants

**Fig. 1:**
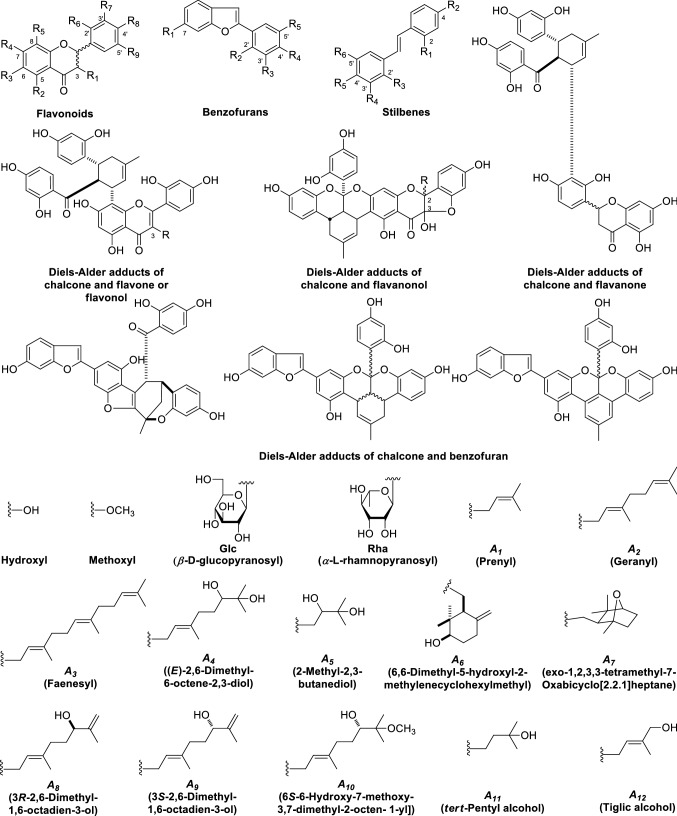
Characteristic structure of compounds from *Morus* genus plants and structural moieties

*Morus* genus plants have a diverse polyphenol profile that includes flavonoids, benzofurans, stilbenes, and Diels–Alder adducts with hydroxyl, methoxyl, glycosyl or prenyl substitution moieties as shown as Fig. 1.

Until now, as one of the main ingredients, more than 190 flavonoids were obtained from the leaves [3, 10, 11] and bark [12, 13] of *Morus* genus plants, which can be grouped into flavone, flavonol, flavanone, flavanonol, and chalcone. Except for chalcones, the hydroxyl and methoxyl groups are usually substituted at C-3, C-5, C-7, C-2′, C-3′ or C-4′, and the glycosylation always occurs at 3-OH. In addition, C-3, C-6, C-8, C-3′ or C-5′ are usually substituted by prenyl and its analogs. Sometimes, the prenyl unit at C-6 and C-8 are cyclized with 5-OH and 7-OH, respectively.

For benzofurans from *Morus* genus plants, the major substitution type is hydroxyl or methoxyl at C-7, C-3′ or C-5′, while the prenyl group substitution often occurs at C-7, C-2′ and C-4′, and the cyclization is always linked between 4′-prenyl and 3′-OH.

Stilbenes, whose chemical structures are similar to those of benzofurans, are another kind of compounds in *Morus* genus plants. The substitution types and positions are similar to those of benzofurans. According to the different numbering rules, the oxygen groups at C-2, C-4, C-3′ and C-5′ and prenyl groups at C-2′ and C-4′ have the most common substitution patterns.

Diels–Alder adducts are another kind of polyphenols in *Morus* genus plants, most of them contain flavonoid groups, and the C-2 and C-3 of the flavonoid unit can be replaced by prenyls and their analogs.

## SARs of bioactive compounds from *Morus* genus plants

As a widely used complementary and alternative medicine, *Morus* genus plants, especially mulberry leaves, were used as adjuvant for blood sugar and TG management, neuroprotection, as anti-tumor agent, immunity regulation, and so on. According to their clinic effects, bioactive compounds in *Morus* genus plants were screened and the SARs to key enzymes partly characterized.

### SARs of α-glucosidase inhibition

Type 2 diabetes is a kind of metabolic disease affecting more and more people all over the world. It is characterized by high postprandial glucose level, high fasting glucose level, insulin resistance, and relative lack of insulin. As one of the effective treatments for high postprandial glucose level, α-glucosidase inhibitors were used to lower the digestion of carbohydrates and reduce the absorption of glucose from the intestine [12, 14]. 1-Deoxynojirimycin and its analogs obtained from leaves of *Morus* genus plants have been considered to be a classical effective α-glucosidase inhibitor [7, 8], and flavonoids, benzofurans and Diels–Alder adducts were also found to play significant roles.

Diels–Alder adducts of chalcones with benzofurans or stilbenes [morusalbins A–D (**1–4**), albanol B (**5**), and mulberrofuran G (**6**) (IC_50_ values: 4.5 ± 0.3, 5.1 ± 0.3, 5.4 ± 0.4, 3.6 ± 0.0, 4.3 ± 0.0, 2.3 ± 0.2, respectively] obtained from the root bark of *M. alba* proved to be more effective than those of flavonoids and benzofurans [albanins A (**7**) and C (**9**), kuwanon C (**8**), albanin T (**10**), moracins M (**11**), S (**12**), mulberrofuran L (**13**) (IC_50_ values: 40.5 ± 5.1, 11.8 ± 0.3, 16.9 ± 2.1, 35.4 ± 0.4, 16.5 ± 1.0, 13.5 ± 1.1, 10.8 ± 0.6 μM, respectively)] on α-glucosidase (enzyme: from yeast; substrate: *p*-nitrophenyl α-d-glucopyranoside) [12].

**Fig. 2:**
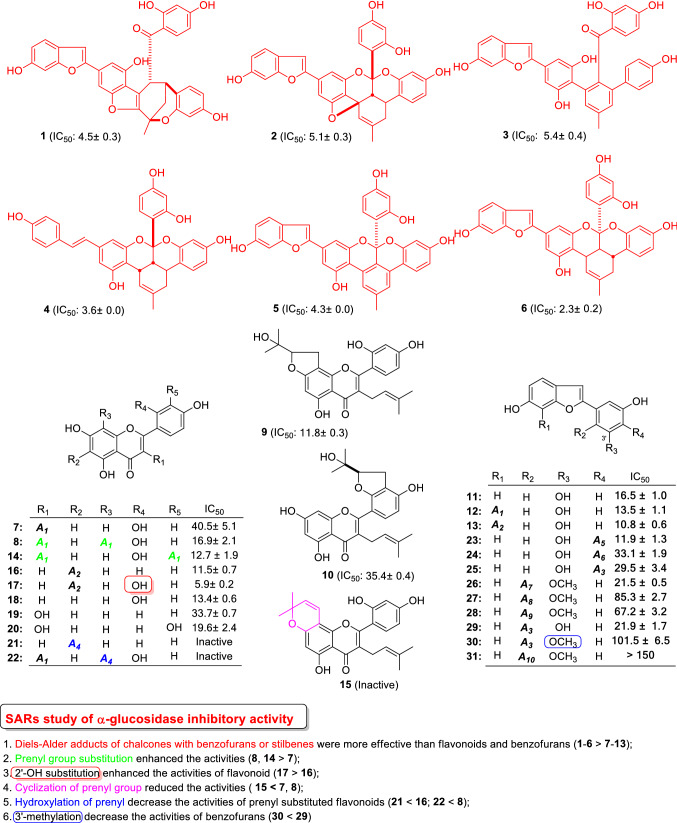
Structures of compounds **1–31** (the structure of ***A***_**1**_***–A***_**10**_ see Fig. 1) for SARs study of α-glucosidase inhibitory activity (the unit of IC_50_: μM)

Comparing the activities of flavonoids, it was suggested that their α-glucosidase inhibitory abilities were influenced by the substitution of prenyl and hydroxyl groups [12, 15]. The SARs were summarized as follows: (1) prenyl group substitution enhanced the activities [kuwanons C (**8**) and T (**14**) vs albanin A (**7**) isolated from the root bark of *M. alba* var. *tatarica*, with IC_50_ values at 16.9 ± 2.1, 12.7 ± 1.9, and 40.5 ± 5.1 μM, respectively)] (enzyme: from yeast; substrate: *p*-nitrophenyl α-d-glucopyranoside) [15]. The cyclization of the prenyl group reduced the activities (**7** and **8** vs **15**) (**15** was inactive) [15]. (2) Hydroxyl substitution located at C-2′ of the flavonoid aglycon could enhance the activity on α-glucosidase inhibition [albanin D (**16**) vs albanin E (**17**) isolated from the root bark of *M. alba* var. *tatarica* with IC_50_ values at 11.5 ± 0.7 and 5.9 ± 0.2, respectively] [15], which could also be proved by the activity determination results of norartocarpetin (**18**), kaempferol (**19**), and quercetin (**20**) from leaves of *M. alba* (IC_50_ values of 13.4 ± 0.6, 33.7 ± 0.7, and 19.6 ± 2.4 μM, respectively) (enzyme: from yeast; substrate: *p*-nitrophenyl α-d-glucopyranoside) [16]. While the hydroxylation of prenyl might decrease the inhibitory activities of prenyl substituted flavonoids [albanin D (**16**, IC_50_ 5.9 ± 0.2 μM) vs mortatarin C (**21**, inactive); kuwanon C (**8**, IC_50_ 16.9 ± 2.1 μM) vs mortatarin B (**22**, inactive) from the root bark of *M. alba* var. *tatarica*] [15] (Fig. 2).

Benzofurans from *Morus* genus plants also showed α-glucosidase inhibitory effect [17]. 4′-(6,6-dimethyl-5-hydroxyl-2-methylenecyclohexylmethyl)-3′,5′,6-trihydroxy-2-arylbenzofuran (**23**), 4′-(6-hydroxy-3,7-di-methyl-2,7-octadien-1-yl)-3′,5′,6-trihydroxy-2-arylbenzofuan (**24**), 4′-(6,7-dihydroxy-3,7-dimethyl-2-octen-1-yl)-3′,5′,6-trihydroxy-2-arylbenzofuan (**25**), 2′-(1,3,3-trimethyl-7-oxabicyclo[2.2.1]hept-2-ylmethyl)-3′-methoxy-5′,6-dihydroxy-2-arylbenzofuran (**26**), (7′'*R*)-2′-(6-hydroxy-3,7-dimethyl-2,7-octadien-1-yl)-3′-methoxy-5′,6-dihydroxy-2-arylbenzofuran (**27**), (7′'*S*)-2′-(6-hydroxy-3,7-dimethyl-2,7-octadien-1-yl)-3′-methoxy-5′,6-dihydroxy-2-arylbenzofuran (**28**), 2′-(6,7-dihydroxy-3,7-dimethyl-2-octen-1-yl)-3′,5′,6-trihydroxy-2-arylbenzofuan (**29**), 2′-(6,7-dihydroxy-3,7-dimethyl-2-octen-1-yl)-3′-methoxy-5′,6-dihydroxy-2-arylbenzofuran (**30**), and 2′-[(6*S*)-6-hydroxy-7-methoxy-3,7-dimethyl-2-octen-1-yl]-3′-methoxy-5′,6-dihydroxy-2-arylbenzofuran (**31**) were isolated from the root bark of *M. alba* var. *tatarica*. The IC_50_ values were 11.9 ± 1.3, 33.1 ± 1.9, 29.5 ± 3.4, 21.5 ± 0.5, 85.3 ± 2.7, 67.2 ± 3.2, 21.9 ± 1.7, 101.5 ± 6.5 and > 150 μM, respectively (enzyme: from yeast; substrate: *p*-nitrophenyl α-d-glucopyranoside). The activity differences between them suggested that the substitution types of prenyl groups were important for α-glucosidase inhibitory effcts. Meanwhile, hydroxyl at C-3′ might be another functional group of benzofurans (**29** vs **30**). The abnormal behavior of **26** might be caused by the effect of the side chain (Fig. 2).

### SARs of lipase inhibition

Hyperlipemia is a high risk factor of obesity, heart disease, diabetes and retinal vascular disease with an abnormally high triglyceride (TG) level in the blood. Pancreatic lipase (PL) is an enzyme that catalyzes triglyceride to fatty acids and glycerol. Inhibition of PL can decrease TG hydrolysis and reduce free fatty acid absorption from intestine to blood and finally lower blood TG level [18, 19]. Orlistat, a PL inhibitor, was used as an anti-obesity drug to inhibit dietary fat absorption and reduce cardiovascular risk factors.

Several flavonoids and benzofurans obtained from *Morus* genus plants were reported to be PL inhibitors. Jeong et al. reported that the PL-inhibitory activities of both flavonols and flavanones from the leaves of *M. alba* would be decreased by the substitution of hydroxyl [kaempferol (**19**) > quercetin (**20**), 7,2′,4′-trihydroxyflavanone (**32**) > steppogenin (**33**)], the activities could also be reduced by glycosylation [quercetin (**20**) > quercetin-3-*O*-glucopyranoside (**34**)] (enzyme: porcine pancreatic lipase; substrate: *p*-nitrophenylbutyrate) [20].

**Fig. 3:**
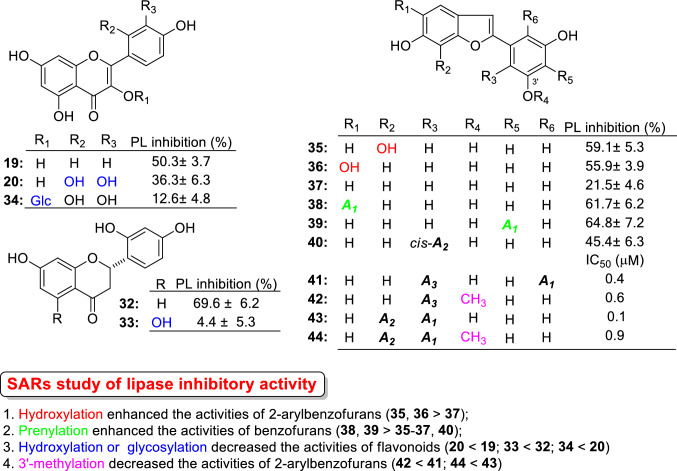
Structures of compounds **19**, **20**, and **32–44** (the structure of ***A***_**1**_***–A***_**3**_ see Fig. 1) for SARs study of lipase inhibitory activity. Relative PL inhibition (%) was calculated as 100—(activity of sample with substrate—negative control of sample without substrate)/(activity of without sample and with substrate—negative control of without sample and substrate) × 100%

For 2-arylbenzofurans from the leaves of *M. alba*, the hydroxylation could enhance the PL-inhibitory activities [2-(3,5-dihydroxyphenyl)-5,6-dihydroxybenzofuran (**35**) and wittifuran E (**36**) > moracin M (**37**)]. Moreover, the prenyl group may also be an active unit in PL inhibition [moracins N (**38**) and C (**39**) > **35***–***37** and albafuran A (**40**)] [20] (Fig. 3). According to Ha et al. report, compounds isolated from the root bark of *M. alba*, morusalfurans B (**41**) and C (**42**), mulberrofuran D (**43**), and sanggenofuran A (**44**) showed inhibitory effect on PL (IC_50_ values: 0.4, 0.6, 0.1, and 0.9 μM, respectively) (enzyme: porcine pancreatic lipase; substrate: *p*-nitrophenylbutyrate) [21]. Through the observation of the structures of bioactive compounds, it was found that if 3′-OH in 2-arylbenzofurans were methylated, their PL inhibitory activity may be decreased (**41** vs **42**, **43** vs **44**) (Fig. 3).

### SARs of tyrosinase inhibition

Tyrosinase is an oxidase distributed in fungi, plants and animals widely. Generally, tyrosinase is known to be involved in melanin synthesis that gives skin, hair, and eyes color. In tumor cells, tyrosinase expression level is significantly up-regulated than in normal cells along with increase of tyrosinase activity. Tyrosinase inhibitors have attracted considerable attention in improving tumor immunity, such as imatinib, an antitumor drug against chronic myelogenous leukemia.

*Morus* genus plants-enriched polyphenol had been used as a kind of non-toxic natural tyrosinase inhibitor to whiten skin [22, 23]. Early in 1998, Shin et al. [24] have studied the tyrosinase inhibitory activity of the hydroxystilbenes isolated from the twig of *M. alba*. The SARs summarized in the reference suggested that the existence of hydroxyl groups in oxyresveratrol and their positions might be important for its inhibitory activity, and the substitution of methyl groups would negatively influence the inhibitory effects. Moreover, Zheng et al. [25] have reported that the substituted position of prenyl/geranyl groups is the key moiety in the tyrosinase inhibitory activity of flavonoids from roots of *Morus nigra*, monoisoprenyl-substituted flavone compounds as well as 2-arylbenzofuran derivatives. Free hydroxyl group may be the active unit, especially for 4′-OH of flavanone, 2- or 4-OH of stilbenes and 4-OH resorcinol skeleton. Intact prenyl group might lead to higher tyrosinase inhibitory activity.

**Fig. 4:**
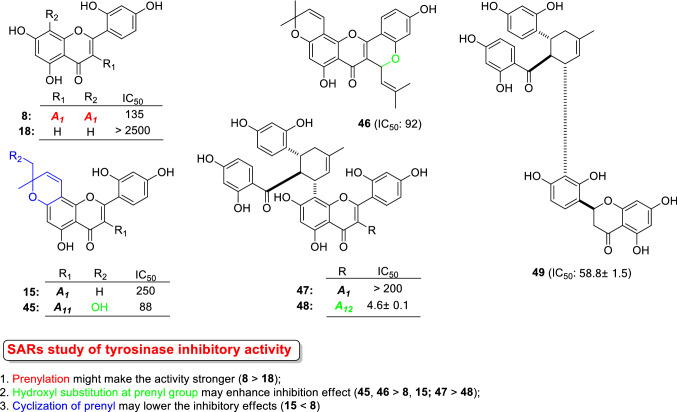
Structures of compounds **8**, **15**, **18**, and **45–49** (for the structure of ***A***_**1**_, ***A***_**11**_, and ***A***_**12**_ see Fig. 1) for SARs study of tyrosinase inhibitory activity (the unit of IC_50_: μM)

Prenyl flavonoids, kuwanon C (**8**), morusin (**15**), norartocarpetin (**18**), mormin (**45**), and cyclomorusin (**46**) isolated from the stem barks of *M. lhou* were found to show tyrosinase inhibitory activities with the IC_50_ values of 135, 250, > 2500, 88, and 92 μM, respectively (enzyme: from mushroom; substrate: l-tyrosine). On comparing their activities, it could be found that the prenyl group might be a crucial active unit (**8** vs **18**), and the cyclization of prenyl may lower the inhibitory effects (**8** vs **15**). Moreover, hydroxyl substitution at the prenyl group leads to enhancing the inhibition effect (**45**, **46** vs **8**, **15**) (enzyme: from mushroom; substrate: l-tyrosine) [26] (Fig. 4).

The comparison of the activities of kuwanon G (**47**) and moracenin D (**48**) with kuwanon L (**49**) from the roots of *M. australis* indicated that the prenyl groups may not be the key active units of a Diels–Alder adduct of chalcone with flavonoid, though the type of prenyl groups would influence the tyrosinase inhibitory activity. When the prenyl group was hydroxylated, a higher inhibition effect appeared (IC_50_ values: > 200, 4.6 ± 0.1, and 58.8 ± 1.5 μM for **47**, **48**, and **49**, respectively) (enzyme: from mushroom; substrate: l-tyrosine) [27] (Fig. 4).

### SARs of β-secretase and acetylcholinesterase inhibition

Alzheimer’s disease (AD) is a neurodegenerative disease that is characterized by memory impairment and loss of recognition, with neuropathological changes such as cerebral β-amyloid angiopathy, neurofibrillary tangles, and glial responses [5, 28].

β-Secretase (BACE) is an integral membrane aspartyl protease that initiates the production of amyloid protein and plays a crucial role in AD occurrence and development. BACE inhibitor can prevent the buildup of β-amyloid and show benefits to AD therapy.

Cho et al. [29] reported the inhibitory effects on human BACE-1 (substrate: oligopeptide) of eight compounds obtained from the stem bark of *M. lhou*, and the SARs suggested that the prenyl groups in flavones and the hydroxyl in ring B played an important role in the BACE-1 inhibitory activities.

A significant feature in the AD brain is the high level of acetylcholinesterase (AChE) associated with β-amyloid plaques. AChE, a key enzyme in cholinergic transmission, promotes the hydrolysis of acetylcholine. AChE inhibitor, such as donepezil, is primarily used to keep acetylcholine levels as high as possible despite cell damage and destruction, to treat memory and learning deficit symptoms.

**Fig. 5:**
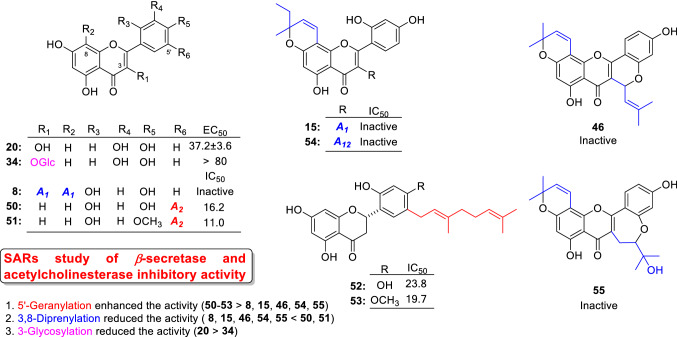
Structures of compounds **8**, **15**, **20**, **34**, **46**, and **50**–**55** (for the structure of ***A***_**1**_, ***A***_**2**_, and ***A***_**12**_ see Fig. 1) for SARs study of β-secretase and acetylcholinesterase inhibitory activity (the unit of EC_50_ and IC_50_: μM)

Four kinds of flavonoids, 5′-geranyl-5,7,2′,4′-tetrahydroxyflavone (**50**) and 5′-geranyl-4′-methoxy-5,7,2′-trihydroxyflavone (**51**), together with kuwanons E (**52**) and U (**53**) obtained from the root bark of *M. lhou* were reported to possess AChE inhibitory activities using acetylthiocholine iodide as substrate, while kuwanon C (**8**), morusin (**15**), cyclomorusin (**46**), morusinol (**54**), and neocyclomorusin (**55**) were inactive [30]. The results indicated that geranyl substituted at C-5′ in the ring B may be crucial for the inhibitory effects of flavonoids or 3,8-diprenylation reduced the activity (Fig. 5). In addition, glycosylation could reduce the protective effects of glutamate-induced oxidative injury in mouse hippocampal HT22 cells [quercetin (**20**) vs isoquercetin (**34**); EC_50_ values: 37.2 ± 3.6, > 80 μM for **20**, **34**, respectively] [31] (Fig. 5).

### Cytotoxicity

**Fig. 6:**
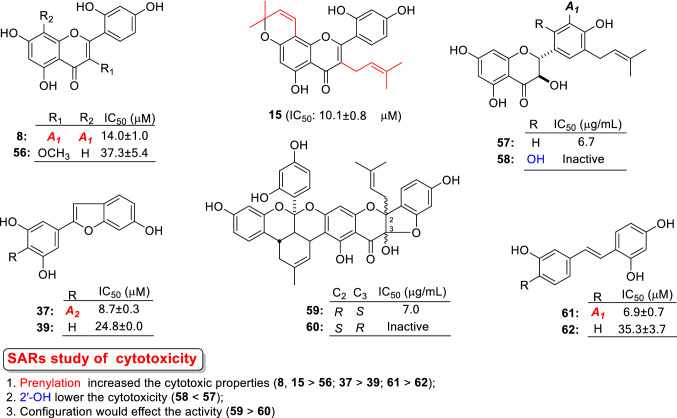
Structures of compounds **8**, **15**, **37**, **39**, **56–62** (for the structure of ***A***_**1**_ and ***A***_**2**_ see Fig. 1) for SARs study of cytotoxicity

Compounds obtained from the *Morus* species, especially prenylated phenols, exhibited significant cell growth inhibition in various cancer cells. The cytotoxic properties against murine leukemia P-388 cells for kuwanon C (**8**), morusin (**15**), and 5,7,2′,4′-tetrahydroxy-3-methoxyflavone (**56**) obtained from the wood of *M. australis* were evaluated [32]. On comparing their inhibitory activities, it was found that the prenyl units at C-7 might be an active unit of prenyl flavonoids (**8**, **15** vs **56** with IC_50_ values at 14.0 ± 1.0, 10.1 ± 0.8, and 37.3 ± 5.4 μM, respectively). In addition, on comparing the activities of compounds cathayanon H (**57**) and cathayanon I (**58**) isolated from the stem bark of *M. cathayana*, it suggested that C-2′ hydroxylation might lower the cytotoxicity [33]. Meanwhile, the configurations of C-2 and C-3 might affect the activity (2*R*3*S* > 2*S*3*R*) [cathayanin B (**59**) vs cathayanin C (**60**, inactivive)] of flavonols (Fig. 6).

For stilbenes and benzofurans from the wood of *M. australis*, the existence of isoprenyls could enhance the inhibitory activities obviously [mulberroside A (**61**) vs oxyresveratrol (**62**) with IC_50_ values at 6.9 ± 0.7 and 35.3 ± 3.7 μM, respectively; moracin C (**37**) vs moracin M (**39**) with IC_50_ values at 8.7 ± 0.3, 24.8 ± 0.0 μM, respectively] [32] (Fig. 6).

## Conclusions

*Morus alba* has a long history of medicinal and edible usage in China. The relatively mature studies on its phytochemistry and pharmacology and clinical trials have led to enormous economic value [3].

On the basis of reports and reviews published, the SARs of α-glucosidase, lipase, tyrosinase, β-secretase, and acetylcholinesterase, and cytotoxicity of compounds obtained from *Morus* genus plants have been summarized. Therefore, prenyl and hydroxyl substituted flavonoids compounds, benzofurans, stilbenes, and Diels–Alder adducts were found to possess significant bioactivities, which may provide some references for the seeking of effective substances with anti-diabetic, anti-obesity, neuroprotective and anti-cancer effects.
