# Correction to: The structure–activity relationship review of the main bioactive constituents of *Morus* genus plants

**DOI:** 10.1007/s11418-020-01406-9

**Published:** 2020-04-10

**Authors:** Jiejing Yan, Jingya Ruan, Peijian Huang, Fan Sun, Dandan Zheng, Yi Zhang, Tao Wang

**Affiliations:** 1 Tianjin State Key Laboratory of Modern Chinese Medicine, Tianjin University of Traditional Chinese Medicine, Nankai District, 312 Anshanxi Road, Tianjin, 300193 China; 2 Tianjin Key Laboratory of TCM Chemistry and Analysis, Institute of Traditional Chinese Medicine, Tianjin University of Traditional Chinese Medicine, Nankai District, 312 Anshanxi Road, Tianjin, 300193 China

## Correction to: Journal of Natural Medicines (2020) 74:331–340 10.1007/s11418-019-01383-8

The article The structure–activity relationship review of the main bioactive constituents of *Morus* genus plants, written by Jiejing Yan, Jingya Ruan, Peijian Huang, Fan Sun, Dandan Zheng, Yi Zhang and Tao Wang was originally published Online First without Open Access. After publication in volume 74 issue 2, page 331–340 the author decided to opt for Open Choice and to make the article an Open Access publication. Therefore, the copyright of the article has been changed to © The Author(s) 2020 and the article is forthwith distributed under the terms of the Creative Commons Attribution 4.0 International License (https://creativecommons.org/licenses/by/4.0/), which permits use, duplication, adaptation, distribution and reproduction in any medium or format, as long as you give appropriate credit to the original author(s) and the source, provide a link to the Creative Commons license, and indicate if changes were made.

The original article was updated.

